# Supplementary figures and images for: Comparison of Preference for Chemicals Associated with Fruit Fermentation between Drosophila melanogaster and Drosophila suzukii and between Virgin and Mated D. melanogaster
Source: Insects. 2023 Apr 14;14(4):382. doi: 10.3390/insects14040382 (PMC10145260; doi:10.3390/insects14040382)

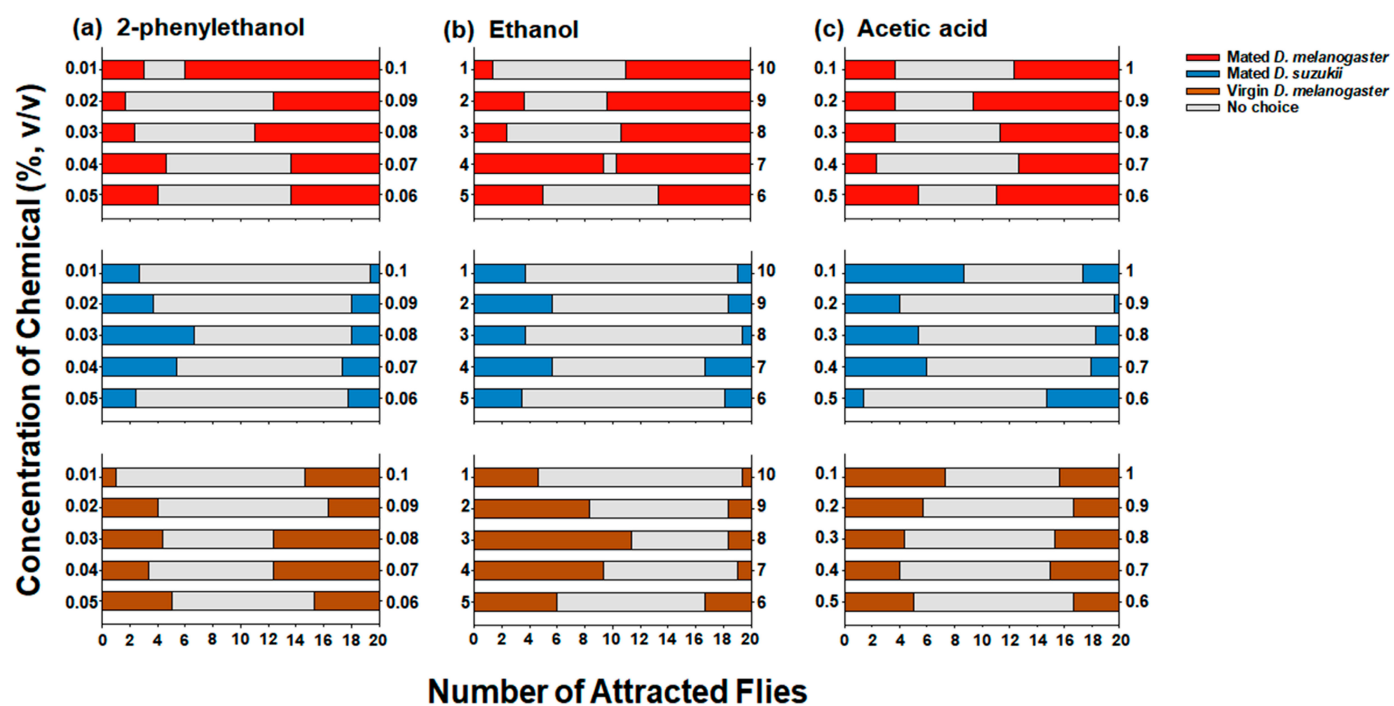

Figure S1. Number of Attracted Flies.

Supplement: Supplementary file 1 [file insects-14-00382-s001.zip › insects-2285017-supplementary.pdf]
